# Chemokines and Chemokine Receptors in the Development of Lupus Nephritis

**DOI:** 10.1155/2016/6012715

**Published:** 2016-06-14

**Authors:** Xiaofeng Liao, Tharshikha Pirapakaran, Xin M. Luo

**Affiliations:** Department of Biomedical Sciences and Pathobiology, Virginia-Maryland College of Veterinary Medicine, Virginia Polytechnic Institute and State University, Blacksburg, VA 24061, USA

## Abstract

Lupus nephritis (LN) is a major cause of morbidity and mortality in the patients with systemic lupus erythematosus (SLE), an autoimmune disease with damage to multiple organs. Leukocyte recruitment into the inflamed kidney is a critical step to promote LN progression, and the chemokine/chemokine receptor system is necessary for leukocyte recruitment. In this review, we summarize recent studies on the roles of chemokines and chemokine receptors in the development of LN and discuss the potential and hurdles of developing novel, chemokine-based drugs to treat LN.

## 1. Introduction

Systemic lupus erythematosus (SLE) is a systemic autoimmune disease with manifestations in multiorgans that are induced by the deposition of circulating autoantibody-autoantigen complexes (immune complexes, IC) and amplified by subsequent infiltration of different types of leukocytes promoting the inflammation [1]. Lupus nephritis (LN), a major cause of morbidity and mortality in up to 60% SLE patients, is characterized by inflammation of the kidney [2]. IC and subsequent complement activation both induce the activation and damage of renal cells that further release inflammatory factors leading to the infiltration of leukocytes into glomerular, tubulointerstitial, and perivascular regions of the inflamed kidney to amplify the renal inflammation and damage [3]. Therefore, leukocyte recruitment to the inflamed kidney is a critical step in the development of LN.

Chemokines are a group of cytokines with small molecular weight whose main action is the recruitment of leukocyte subsets under homeostatic and pathological conditions. Through interacting with chemokine receptors that are expressed on the cell surface as 7-transmembrane proteins coupled with G-protein for signaling transduction, chemokines can induce firm adhesion of targeted cells to the endothelium and direct the movement of targeted cells to their destination according to the concentration gradient of a given chemokine [4]. Through this mechanism chemokines can induce directed chemotaxis of responsive cells. Chemokines are classified into four subfamilies according to the first two cysteines and the amino acid residues in between at N-terminal end of the polypeptide. Based on whether the first two cysteines are adjacent, separated by one residue, or separated by three residues, a chemokine is classified into CCL, CXCL, or CX3CL family, respectively. If lacking two first cysteines, the chemokine belongs to XCL family. Chemokine receptors are named corresponding to the subfamilies of chemokines as CCR, CXCR, CX3CR, or XCR, respectively. Individual chemokines and chemokine receptors discovered to date have been reviewed elsewhere with summarized tables [5–8]. They are important both at steady state and during inflammation. Homeostatic chemokines and chemokine receptors are those important for the homing of progenitor cells and mature immune cells into the primary/secondary immune tissues for the development of the immune system and into peripheral nonimmune tissues for the tissue-specific functions and immune surveillance [5, 9]. Examples are CCR7 that is expressed on naïve lymphocytes and dendritic cells for recruitment into lymph nodes by CCL19 and CCL21 as part of the normal immune system development; CXCL12 that is important for the retention of CXCR4^+^ hematopoietic stem cells (HSCs) in HSC niches in the bone marrow; CCL2 that is critical for CCR2^+^ monocytes emigration from the bone marrow; and CX3CL1 that is essential for CX3CR1^high^ monocytes patrolling along the blood vessels. On the other hand, when there is an infection or injury-induced inflammation, activated immune cells will upregulate some chemokine receptors and migrate into inflamed immune and nonimmune tissues by recognizing correspondingly increased inflammatory chemokines. For example, upon the stimulation of pathogen-associated molecular pattern molecules (PAMP) or danger-associated molecular pattern molecules (DAMP), resident mast cells, macrophages, and dendritic cells will release cytokine signals to induce the upregulation of several inflammatory chemokines expressed by activated endothelial and epithelial cells, such as CCL2, CCL3, CCL4, CCL5, CXCL1, CXCL2, CXCL3, CXCL5, and CXCL8. Consequently, circulating immune cells such as neutrophils, monocytes, and effector T cells will migrate into inflamed tissues using related chemokine receptors such as CCR2, CCR1, and CXCR2.

Chemokines, unlike adhesion molecules broadly shared by different types of immune cells, are selectively used by specific cell populations and have been found to be involved in the migration of leukocytes to nephritic kidney of both SLE patients and lupus-prone mice. Studies have shown that several immune cell populations are accumulated in the kidney in LN, including various T cell subsets, B cells, plasma cells, NK cells, monocytes/macrophages, dendritic cells (DC), and neutrophils [10, 11]. In this review, we summarize recent studies on the chemokines that are increased in the kidney as LN progresses and the corresponding chemokine receptors used by renal-infiltrating leukocytes in response to the chemokines. We focus on those highlighted by multiple studies, including the chemokines CXCL13, CXCL12, CXCL9, CXCL10, CXCL11, CCL2, CCL3, CCL4, CCL5, and CX3CL1 and chemokine receptors CXCR5, CXCR4, CXCR3, CCR1, CCR2, CCR5, and CX3CR1. Inflammatory factors inducing the expression of chemokines, as well as chemokines that may be used as biomarkers for the diagnosis of LN, are also discussed.

## 2. Key Chemokines and Chemokine Receptors in LN

Some chemokines are more commonly related to LN than others based on both studies of lupus-prone mouse models and SLE patients, which have been summarized in Tables 1 and 2, respectively. To interact with specific chemokine receptors expressed on particular cell populations, these chemokines have diverse biological effects by influencing the migration of different cell populations in both healthy and disease situations. In LN disease, animal model studies suggest that these chemokines contribute to systemic autoimmune responses in immune tissues—thus indirectly promoting LN—and are involved in local renal inflammation with direct effects. Evidence from human studies, on the other hand, suggests the clinical involvement of these chemokines in the development of LN. In the following sections we will discuss each of these LN-related chemokines regarding their biological effects, roles in mouse models of LN, and clinical evidence from studies of lupus nephritic patients.

### 2.1. CXCL13 and CXCR5

CXCL13, also known as B cell-attracting chemokine 1 (BCA1) or B lymphocyte chemoattractant (BLC), is the chemokine recognized by CXCR5. CXCL13 is important in directing the trafficking of CXCR5^+^ cells, including B cells, follicular helper CD4 T cells (T_FH_), CXCR5^+^CD8^+^ T cells, and CXCR5^+^ DC, all involved in humoral immune responses [12–14]. Both B cells and T_FH_ critical for the formation of germinal centers (GC) depend on CXCR5 to migrate into the B cell follicles in secondary immune tissues [12, 15–17]. Circulating CXCR5^+^ central or effector memory-like T_FH_ have also been discovered that could migrate to and function in immune and nonimmune tissues [18–21]. Besides T_FH_, CXCR5^+^CD8^+^ T cells and CXCR5^+^ DC are also found to facilitate B cell responses [13, 14]. Therefore, by attracting different types of CXCR5^+^ immune cells, CXCL13 contributes to B cell responses, especially the generation of high affinity antibody-producing cells in GC.

In two commonly used lupus-prone mouse models, NZB/W F1 and MRL/lpr, transcript levels of renal CXCL13 and CXCR5 are consistently increased in aged lupus nephritic mice compared to nonlupus control mice or young mice prior to disease onset [22–25], suggesting their involvement in the development of LN. Renal macrophages and DC in lupus-prone mice may be the source of CXCL13 in the nephritic kidney [24, 26–29], leading to increased migration of CXCR5^+^ B cells and T_FH_-like cells into the inflamed kidney towards CXCL13 [24, 26, 30]. Further studies with NZB/W F1 mice have shown that, among B cell populations, B1 cells compared to B2 cells express much higher CXCR5 and migrate towards CXCL13 more efficiently* in vitro* [22, 30], and preferentially migrate into the kidney and lung of diseased mice instead of lymphoid tissues [30]. Functionally, B1 cells isolated from NZB/W F1 mice, but not B2 cells, can activate T cells in allogeneic mixed lymphocyte reaction. CXCR5^+^ CD4^+^ T cells, on the other hand, have been shown to promote IgG production from B1 cells* in vitro*, suggesting potential interaction of B1 and T cells* in situ* in the nephritic kidney [30]. However, another study found most renal-infiltrating B cells to be non-class-switched B2 cells in NZB/W F1 mice [24], leaving the role of renal CXCR5^+^ B in LN an open question. While further studies are required, these results suggest important functions of CXCR5 and its ligand in the development of LN.

The critical roles of CXCL13 and CXCR5^+^ cells in the pathogenesis of LN are also evidenced by studies of anti-CXCL13 neutralizing antibodies in MRL/lpr lupus-prone mice and CXCR5-deficiency in B6/lpr lupus-prone mice [31, 32]. Renal pathology, including proteinuria and serum creatinine levels, glomerular and perivascular scores, deposition of IC and complement C3, and renal IL-1*β*, IL-6, IL-33, and IL-17 protein levels, was significantly lower in the neutralizing antibody-treated mice than controls [31]. Systemic autoimmune responses such as the level of circulating anti-double stranded DNA (anti-dsDNA) antibodies and the ratio of splenic Th17/Treg were reduced as well, suggesting that the pathogenic role of CXCL13/CXCR5 may not be kidney-specific. As the administration of anti-CXCL13 neutralizing antibodies is not tissue-specific, it is difficult to say if reduced renal pathology is due to the secondary effect of decreased systemic autoimmune responses or the direct effect of blocking CXCL13/CXCR5 signal in the kidney. Similar to the CXCL13 blockade study, CXCR5-knockout in B6/lpr mice also downregulated systemic autoimmune reactions, including reduced lymphadenopathy and splenomegaly with reduced GC, B cells, plasma cells, and double negative (DN) T cells in secondary lymphoid organs, as well as reduced circulating IgG [32]. Importantly, this study also showed reduced infiltration of adoptively transferred DN T cells from CXCR5-deficient B6/lpr mice compared to wild type B6/lpr mice into the kidney of Rag1^−/−^ recipient mice, indicating direct contribution of CXCL13/CXCR5 signal to local renal inflammation in LN. Therefore, CXCL13/CXCR5 contributes to the development of LN both systemically in immune tissues and locally in the kidney of lupus-prone mice.

Evidence from the studies of SLE patients with LN further suggests the clinical involvement of CXCL13/CXCR5 in the development of LN. In SLE patients with LN, but not in healthy controls (HC), CXCL13 and CXCR5 are highly expressed in the cortex of the kidney [33]. In addition, B cells and T_FH_-like cells that express CXCR5 have been indicated to infiltrate the nephritic kidney of SLE patients and are colocalized with CXCL13-expressing regions [34]. Besides chemoattractant functions, CXCL13 may also contribute to LN by activating CXCR5^+^ renal nonimmune cells such as human podocytes to produce proinflammatory molecules [35].

### 2.2. CXCL12 and CXCR4

CXCL12, also known as stromal cell-derived factor 1 (SDF-1), is the ligand of chemokine receptor CXCR4. It is involved in the homing, retaining, and survival of CXCR4^+^ hematopoietic stem cells, B cell precursors, and plasma cells in the bone marrow. In addition, CXCL12 maintains the homeostasis of neutrophils, T cells, and B cells in immune and nonimmune tissues [36–38].

Studies have shown possible involvement of CXCL12 and CXCR4 in LN development. In lupus-prone mouse models, CXCR4 is consistently increased in various immune cell populations including B cells, plasma cells, T cells, neutrophils, and monocytes in the circulation and spleen of diseased mice, suggesting enhanced chemotaxis of these cells towards CXCL12 [39–41]. Importantly, both CXCL12 and CXCR4 expressions are increased in the kidney of diseased lupus-prone mice, indicating migration of CXCR4^+^ cells into the kidney as LN progresses [23, 24, 40, 42]. In particular, studies have shown the increased accumulation of CXCR4^+^ cells in the kidney of diseased mice, including plasma cells, Foxp3^+^ CD4^+^ regulatory T (Treg) cells, Foxp3^−^ CD4^+^ conventional T cells, and inflammatory monocytes and neutrophils [40, 41, 43]. Besides promoting leukocyte infiltration, CXCL12/CXCR4 may also deteriorate LN by directly affecting renal tissue cells. Studies have shown that activated parietal epithelial cells (PECs) as glomerular progenitor cells are involved in proliferative glomerulonephritis [44]. Normally, PECs possess regenerative potential for the repair of injured kidney [45, 46]. However, in glomerulonephritis, CXCR4 is overexpressed on PECs upon inflammatory stimulation, whereas autoantibodies and inflammatory mediators stimulate CXCL12 production on injured podocytes [42, 44, 47]. Consequently, through the interaction between CXCL12 and CXCR4, PECs migrate into the glomerular tuft during the development of LN, where they predominately form hyperplastic lesions in proliferative glomerulonephritis and lead to glomerulosclerosis by secreting extracellular matrix [3, 44].

Blocking the interaction of CXCL12/CXCR4 in lupus-prone mice reveals their contributions to both systemic autoimmune responses in secondary lymphoid organs and local renal inflammation. Administration of anti-CXCL12 neutralizing antibodies in NZB/W F1 mice led to increased survival rate and reduced renal inflammation including decreased proteinuria and IgG deposition [42]. This may be at least partially due to decreased systemic autoimmune reactions, since circulating anti-dsDNA IgG and B1a subset in the peritoneal cavity and the spleen were reduced, as well as reduced activated CD4^+^ T cells in the spleen and lymph nodes. The direct role of CXCL12/CXCR4 network to recruit immune cells in the lupus nephritic kidney is demonstrated in another study with administration of a CXCR4 antagonist in B6.SleYaa lupus-prone mice [40]. Similar to anti-CXCL12 neutralizing antibodies, CXCR4 antagonist ameliorated LN with decreased renal pathological scores and proteinuria and prolonged lifespan. Early administration before severe proteinuria also led to reduced splenomegaly and circulating ANA IgG, suggesting a systemic effect. Splenic monocytes, activated T cells, and B cells in marginal zone and follicular and germinal center were similarly reduced. However, late administration after the onset of severe proteinuria did not influence systemic autoimmune responses but led to reduced infiltration of monocytes, neutrophils, and CD4^+^ T cells into the kidney, suggesting a direct effect of CXCL12/CXCR4 in the kidney.

In patients with LN, it has been consistently demonstrated that CXCL12 expression is significantly increased in tubules and glomeruli of the kidney [62], while most circulating CD4^+^ T cells and B cells express CXCR4 in SLE patients [62–65]. Although it is debatable whether its level on B cells is reduced [64] or increased [62, 65], CXCR4-expressing B cells are found to be accumulated in the renal biopsy samples of patients with LN [65], suggesting involvement of CXCL12/CXCR4 in the kidney of patients with LN.

### 2.3. CXCL9/10/11 and CXCR3

CXCR3 is a chemokine receptor interacting with three interferon-inducible chemokines, CXCL9 (monokine induced by gamma-interferon, MIG), CXCL10 (interferon-induced protein of 10 kDa, IP-10), and CXCL11 (interferon-inducible T cell alpha chemoattractant, I-TAC) [72]. Several immune cell populations have been reported to express CXCR3, including NK cells, plasmacytoid DC (pDC), conventional DC (cDC), B cells, and activated T cells [73]. Among activated T cells, T helper 1 (Th1) cells and effector CD8^+^ T cells preferentially express CXCR3. T helper 17 (Th17) cells have also been reported to express CXCR3, although CCR6 is the predominant chemokine receptor on their surface. Since the expression of CXCR3 is induced upon activation of immune cells, especially in effector T cell populations, activated immune cells can migrate into inflamed peripheral tissues where CXCR3 ligands are induced. The three CXCR3 ligands under different circumstances have shown redundancy, dominance, collaboration, or antagonism to one another [72]. Therefore, CXCR3 and its ligands are mainly associated with the effector stage of immune response and are regulated in a more complex fashion than single paired chemokines/chemokine receptors.

CXCR3 and its ligands are involved in the pathogenesis of SLE. In lupus-prone mice, most commonly NZB/W F1 mice and MRL/lpr mice, CXCR3-expressing T cells and plasma cells as activated effector populations in the secondary lymphoid organs are increased during the development of LN [41, 48, 66, 74]. Importantly, studies have shown that CXCR3 and its ligands are increased in the nephritic kidney of lupus-prone mice, suggesting migration of CXCR3-expressing effector cells from the secondary lymphoid organs into the inflamed kidney [23, 24, 49, 66, 74–76]. Detailed studies with MRL/lpr and NZB/W F1 mice reveal that CXCR3 is expressed on different renal-infiltrating cells with varied proportions, including CD4^+^ T cells (15–33%), CD8^+^ T cells (10–33%), B220^+^ cells (including both B cells and pDC, 25%), plasma cells (40%), and macrophages (5%) [23, 48, 49]. All renal CXCR3^+^ T cells are confirmed as CD44^+^ activated T cells, while CD44^−/low^ naïve T cells are CXCR3-negative [48]. In addition, renal-infiltrating CXCR3^+^ plasma cells can secrete IgG instead of IgM, indicating their pathogenic role in promoting LN [74].

While both CXCR3 and its ligands are increased in the kidney of lupus-prone mice with LN, their deficiencies in lupus-prone mice have shown inconsistent or even contradictory results. CXCL10 deficiency in MRL/lpr mice showed no change of LN severity [23]. However, CXCR3- or CXCL9-deficiency in the nephrotoxic serum nephritis (NSN) model showed reduced nephritic disease with decreased IgG deposits and activated T cells and macrophages in the kidney [23]. This suggests that CXCL9 rather than CXCL10 may be critical for CXCR3-dependent cellular infiltration of the kidney in LN. Consistent with this, another study showed that CXCL9 in the kidney of diseased MRL/lpr mice was the most abundant chemokine for T cell trafficking [49]. However, circulating antigen-specific IgG was also reduced in CXCR3- or CXCL9-deficient NSN model, suggesting that CXCR3/CXCL9 interaction may influence systemic immune responses and indirectly affect kidney pathology [23]. Further studies with CXCR3-knockout MRL/lpr and NZB/W F1 mice have shown different effects on the development of LN. With CXCR3-deficiency in MRL/lpr mice, glomerular pathology score was reduced with decreased T cells and macrophages infiltration around glomeruli, ameliorated renal lesion, and decreased proteinuria [66]. IFN*γ*-producing T cells and IL-17-producing T cells were also reduced in the kidney but not in the spleen or lymph nodes of CXCR3-knockout MRL/lpr mice. Importantly, renal IgG and C3 deposits and circulating total IgG and anti-dsDNA IgG were not different between CXCR3-knockout and wild type MRL/lpr mice, suggesting a direct effect of CXCR3 and its ligands on the kidney by recruiting activated effector T cells and macrophages. However, in NZB/W F1 mice, CXCR3 deficiency did not change either the infiltration of plasma cells and T cells to the kidney or the course of LN [48]. Therefore, further studies are required to determine whether CXCR3 is important for LN development and which ligand(s) are critical for the infiltration of CXCR3^+^ cells to the kidney of lupus-prone mice.

Despite controversial results from studies of lupus-prone mice, evidence from SLE patients still suggests the possible involvement of CXCR3 and its chemokine ligands in the development of LN. Patients with active SLE compared to HC or patients with inactive disease have reduced CXCR3^+^ CD4^+^ T cells in the circulation, suggesting infiltration of the cells into peripheral tissues [63]. In addition, several studies have shown that, in SLE patients with LN, compared to HC or patients without nephritic involvement, CXCR3^+^ cells (mostly T cells) are increased in the kidney and urine, which is correlated with increased expression of renal CXCR3 ligands [26, 66, 67]. Moreover, it has been found that CXCR3^+^ cells are accumulated in tubulointerstitial regions and around glomeruli in the kidney of lupus nephritic patients [26], account for 60% of total infiltrating cells, and are positively correlated with proteinuria [67]. Among the three CXCR3 ligands, CXCL10 is most increased in SLE patients and localized in the same region as CXCR3^+^ cells in the nephritic kidney [67]. Besides CXCR3-expressing T cells, a group of pathogenic CD19^high^ B cells also express CXCR3 at a high level in SLE patients and migrate towards CXCL9* in vitro*, suggesting their potential to migrate into inflamed peripheral tissues such as the kidney [68].

### 2.4. CCR1, CCR5, and CCL3/4/5

CCR1 and CCR5 share the same ligands, CCL3 (macrophage inflammatory protein 1-alpha, MIP-1*α*) and CCL5 (regulated upon activation, normally T-expressed, and presumably secreted, RANTES). CCR5 also responds to CCL4 (macrophage inflammatory protein 1-beta, MIP-1*β*). CCR1 is expressed on CD34^+^ bone marrow progenitor cells, monocytes, NK cells, T cells, and preferentially on CD45RO^+^ activated/memory T cells [50, 77]. Murine neutrophils also express CCR1 [78]. CCR5 is expressed on monocytes/macrophages and T cells (both CD4^+^ and CD8^+^ subsets), especially on Th1 cells [50, 79]. Interestingly, monocytes express a high level of CCR1 but low CCR5, while the expression pattern is the opposite in activated/memory T cells, suggesting selective expression of CCR1 and CCR5 in monocytes and activated T cells, respectively [80].

CCR1 or CCR5 knockout mice have been developed to study their functions in different disease models [50]. Even though CCR1 and CCR5 share the same chemokine ligands, studies with unilateral ureteral obstruction and renal ischemia-reperfusion injury models have shown that CCR1 but not CCR5 is essential for T cells, macrophages, and neutrophils infiltration in the tubulointerstitial region of the kidney [81–83]. Moreover, CCR1-deficient mice have enhanced macrophage and T cell infiltration to the glomerular region of the kidney in a nephrotoxic nephritis model, suggesting that such infiltration is CCR1-independent [84]. CCR1-deficient mice also exhibit increased circulating antigen-specific, Th1-biased, pathogenic IgG2a response, indicating that CCR1 is also involved in Th1-dependent systemic humoral immune response [84]. However, in a host versus graft disease (HVGD) model, CCR1-deficiency shows a protective effect by inhibiting chronic cardiac allograft rejection [85], which makes the role of CCR1 complicated in different diseases possibly depending on whether humoral immune responses are involved and/or which tissues and immune cell populations are involved. Regarding CCR5 deficiency, macrophages from CCR5-knockout mice have reduced ability to produce inflammatory cytokines including IL-6, IL-1*β*, and TNF*α*, rendering defective bacteria clearance in a* Listeria monocytogenes* infection model [50]. CCR5-deficient T cells, on the other hand, have elevated production of IFN*γ*, granulocyte macrophage colony-stimulating factor (GM-CSF), and IL-4 with enhanced delayed-type hypersensitivity reaction and humoral immune responses following antigen challenge in CCR5-deficient mice [50]. CCR5 also contributes to the recruitment of Treg in lymphoid and nonlymphoid tissues, which is important in suppressing effector responses in graft versus host disease- (GVHD-) targeted organs [50]. Therefore, CCR5 deficiency in different diseases leads to different outcomes depending on which cell types are critical and whether the initial immune response (in lymphoid organs) or the effector phase (in nonimmune tissues) is involved [50].

In lupus-prone mice, CCR1, CCR5, and their ligands are increased in the kidney during LN development [24, 25, 28, 49–52, 54, 75, 76]. Studies have shown that, in nephritic NZB/W F1 mice, both renal T cells and monocytes/macrophages have elevated CCR1 expression on the surface [50]. In MRL/lpr mice, the administration of a CCR1 antagonist at late stage improved LN with reduced interstitial lesions including decreased infiltration of T cells and monocytes/macrophages, reduced inflammation-induced proliferating and apoptotic cells, and reduction of tubular atrophy and interstitial fibrosis [26]. However, glomerular IgG deposits and different isotypes of circulating anti-dsDNA IgG reflecting systemic humoral autoimmune response did not change, suggesting a direct effect of CCR1 antagonism on preventing renal infiltration of T cells and macrophages. This was confirmed by reduced renal infiltration of adoptively transferred T cells and macrophages pretreated with the CCR1 antagonist. The role of CCR1 in LN was limited in interstitial region, as glomerular injury and proteinuria were not improved by CCR1-antagonist administration in MRL/lpr mice [26]. In NZB/W F1 mice, the effect of CCR1 antagonist administration at late stage has also been studied. Besides the similar effects of CCR1 blocking T cells and macrophage infiltration, the study with NZB/W F1 mice also showed prolonged lifespan and improved glomerular injury including reduced proteinuria [50].

In MRL/lpr mice, the extent of CCR5 expression is debated, as over 50% of renal T cells express CCR5 in one study, whereas only 1% of T cells are shown to express CCR5 in another study [49, 52]. Renal-infiltrating macrophages, on the other hand, are CCR5-positive in MRL/lpr mice [52]. Contrary to the effects of CCR1 blocking, CCR5 knockout in MRL/lpr mice deteriorated LN with increased proteinuria and tubulointerstitial infiltration of total CD3^+^ T cells and F4/80^+^ macrophages in the kidney [53]. Foxp3^+^ Tregs were also increased in the kidney of CCR5-knockout MRL/lpr mice, but LN progression was not reversed by the increase of Treg cells. Systemic humoral immune responses were not affected by CCR5 deficiency, as the circulating anti-dsDNA IgG and renal IgG/C3 deposits were not different between CCR5-knockout and wild type MRL/lpr mice. However, CCR5-knockout MRL/lpr mice exhibited increased splenomegaly and elevated circulating/renal CCL3, suggesting that renal-infiltrating immune cells may use alternative chemokine receptors responding to CCL3 such as CCR1 to migrate into the kidney and promote LN. This study reveals possible roles of CCR5 in negatively regulating LN progression by modulating CCL3 production and controlling lymphoproliferation in the spleen [53]. Therefore, it appears that CCR1 and CCR5 may, respectively, promote or attenuate the development of LN in lupus-prone mice.

In SLE patients, CCR1, CCR5, and their ligands are also increased in the kidney during the development of LN [69, 70, 86, 87]. Evidence from SLE patients further shows that most CCR1^+^ cells infiltrating in the kidney are CD68^+^ macrophages [63, 69], while CCR5, on the other hand, is expressed on both circulating and renal-infiltrating T cells in SLE patients, particularly interstitial infiltrating T cells [70].

### 2.5. CCL2 and CCR2

CCR2 is expressed on a fraction of monocytes, dendritic cells, NK cells, and T cells, and one of its ligand is CCL2 (monocyte chemoattractant protein-1, MCP-1) [50, 88]. CCR2 expression on monocytes is important for both their egression from bone marrow and extravasation into inflamed tissues [50]. Besides chemoattractant function for monocytes, CCR2 and CCL2 are also involved in the regulation of T cell activation and differentiation. T cells from CCR2-deficient mice produce less IFN*γ* upon stimulation [50], while CCL2 is associated with Th2 cell polarization and enhances IL-4 production by T cells [88].

In lupus-prone mice, CCR2 and CCL2 are increased in the kidney during the development of LN, suggesting the recruitment of CCR2^+^ leukocytes into the inflamed kidney by CCL2 [24, 25, 54–56, 89]. Using MRL/lpr mice, studies have shown that most renal-infiltrating CCR2^+^ cells are macrophages and not T cells [52, 54]. In addition, CCL2 is mainly expressed in the tubulointerstitial regions of the kidney in lupus-prone mice [55, 89].

By blocking the interaction between CCR2 and CCL2 in MRL/lpr lupus-prone mice, studies have shown that CCL2/CCR2 network contributes to LN development through both systemic and local mechanisms. In both CCL2/CCR2 antagonist experiments and CCL2/CCR2-knockout models, animal lifespan was consistently prolonged with reduced LN including less glomerular and tubulointerstitial infiltration of T cells and macrophages, although severe proteinuria in old mice was not improved [55–59]. In addition, the pathology and inflammation in the lung and skin of CCL2/CCR2-deficient MRL/lpr mice were reduced, suggesting the systemic involvement of CCL2/CCR2 in multiperipheral tissues. By further comparing the differences between antagonist and knockout models, it was evident that CCL2/CCR2 antagonists did not improve splenomegaly, lymphadenopathy, and circulating total/autoantibodies, suggesting the local involvement of CCL2/CCR2 in autoimmune target tissues, such as the kidney [56, 58, 59]. In contrast, CCL2/CCR2-knockout MRL/lpr mice exhibited reduced circulating anti-dsDNA IgG, diminished lymphadenopathy, and decreased percentage of circulating CD8^+^ T cells, suggesting CCL2/CCR2 network also contributes to systemic autoimmune reactions in the immune tissues, through which LN progression was indirectly promoted [55, 57]. Interestingly, anti-CCL2 spiegelmer, a CCL2 antagonist, blocked the emigration of monocytes from the bone marrow of MRL/lpr mice, which suggested an additional mechanism of how CCL2/CCR2 may promote LN by facilitating monocytes migration from the bone marrow into the kidney [59]. Together, these results suggest the importance of CCR2 and CCL2 in promoting LN.

In SLE patients, CCR2 and CCL2 expression is also increased in the kidney during the development of LN [77]. Same as shown in lupus-prone mice, CCL2 is mainly expressed in the tubulointerstitial region of the kidney in SLE patients [77]. Renal endothelial cells, epithelial cells, and infiltrating leukocytes could be the source of CCL2 [77]. In patients with active SLE, a small proportion of T cells (both CD4^+^ and CD8^+^) express CCR2 and are reduced in the blood circulation, suggesting their migration from the blood to inflamed peripheral tissues such as the kidney [63].

### 2.6. CX3CL1 and CX3CR1

CX3CL1, also known as fractalkine, is the only chemokine with CX3C-motif discovered to date that interacts with its unique chemokine receptor, CX3CR1. CX3CR1 is expressed on a fraction of monocytes/macrophages, dendritic cells, NK cells, T cells, and particularly CD8^+^ cytotoxic T cells [90–93]. Different from other chemokines, CX3CL1 possesses a soluble form and a transmembrane form, which function to induce chemotaxis and adhesion of CX3CR1^+^ leukocytes, respectively. CX3CR1/CX3CL1 interaction also provides antiapoptotic signals to sustain the survival of CX3CR1^+^ leukocytes [50, 94, 95].

Studies have shown possible involvement of CX3CL1 and CX3CR1^+^ leukocytes in the development of LN. In MRL/lpr mice, CD16^+^ cells in glomeruli are increased with lupus development [60], with increased protein level of CX3CL1 detectable in glomeruli, interstitial microvasculature, and arterial regions [61]. Unlike MRL/lpr mice, CX3CR1 and CX3CL1 expression in the kidney of NZB/W F1 mice do not change with lupus progression, suggesting differences between various lupus-prone mouse models [23, 24, 61, 96].

Administration of NH2-terminally truncated CX3CL1 analogs blocked CX3CL1/CX3CR1 interaction and significantly ameliorates glomerular and vascular lesions in MRL/lpr mice, reducing the infiltration of macrophages and CX3CR1^+^ cells to the glomerular, interstitial, and perivascular regions [61]. T cells, however, were only reduced in the interstitial regions. With CX3CR1 blockade, the transcription level of renal IFN*γ* and IL-2 was reduced as well. The levels of circulating anti-dsDNA IgG and IgG-containing IC were otherwise not affected, which, together with unchanged splenomegaly and lymphadenopathy, suggested a direct function of CX3CL1/CX3CR1 in the kidney that promotes LN progression in MRL/lpr mice [61].

In SLE patients, CX3CL1 expression is significantly increased in the glomeruli in class IV glomerulonephritis compared to other classes [71]. In addition, glomerular CX3CL1 expression is positively correlated with the infiltration of glomerular CD16^+^ cells that express CX3CR1, which deteriorates lupus disease, suggesting the clinical involvement of CX3CL1/CX3CR1 in LN development.

### 2.7. Other T Helper Cell-Associated Chemokine Receptors/Chemokines

Aside from the T cell-related chemokine receptors discussed above, CXCR6, CCR4, and CCR6 that are associated with the recruitment of Th1, Th2, and Th17/Treg, respectively, have been also studied in SLE [16, 97, 98].

CXCR6 and its ligand CXCL16 have been shown to be involved in autoimmune diseases such as rheumatoid arthritis [99]. Blockade of CXCL16 in mice also attenuates glomerulonephritis induced by antiglomerular basement membrane antibodies [100]. In both MRL/lpr and NZB/W F1 mice, the expression of CXCR6 and CXCL16 is increased during the development of LN [24, 75]. While the lack of available blocking antibodies has hindered the investigation of the role of CXCR6/CXCL16 in LN, CXCR6 has been shown to facilitate the infiltration of activated CD8^+^ T cells to the inflamed liver [101]. It is thus possible that CXCR6^+^ CD8^+^ T cells may be recruited into the inflamed kidney in LN through a CXCL16-dependent mechanism. Moreover, it has been shown that the level of soluble CXCL16 (sCXCL16) in the serum of SLE patients is significantly higher compared to HC and is positively correlated with SLE disease activity index (SLEDAI) of patients [102]. In addition, the concentration of sCXCL16 drops with disease remission. Therefore, CXCL16 may be involved in LN development by recruiting CXCR6^+^ T cells into the nephritic kidney.

CCR4 has two chemokine ligands, CCL17 (thymus and activation-regulated chemokine, TARC) and CCL22 (macrophage-derived chemokine). The expression of renal CCR4 and its two ligands is increased in MRL/lpr mice as LN progresses [23]. Interestingly, blockade of CCL22, but not CCL17, in MRL/lpr mice led to reduced proteinuria and serum creatinine with improved renal function [56, 103]. Moreover, the number of CCR4^+^ T cells is reduced in the peripheral blood of SLE patients compared to HC, suggesting increased migration of these cells into inflamed tissues [87]. Accordingly, CCR4^+^ cells are found in the kidney of SLE patients that colocalize with CD4^+^ cells. Thus, CCR4^+^ T cells may selectively use CCL22 to migrate into the lupus nephritic kidney.

CCR6 is the chemokine receptor for CCL20 (liver and activation-regulated chemokine, LARC, or macrophage inflammatory protein 3*α*, MIP-3*α*). CCR6 is expressed on T cells, preferentially on Th17 and Treg cells [40, 104–110]. The interaction of CCR6 and CCL20 can recruit Treg and Th17 cells into the kidney in murine nephrotoxic nephritis [98, 111]. Whether this interaction can recruit Th17 cells into the kidney to promote LN, or recruit Treg cells to attenuate LN, remains to be explored. In NZB/W F1 mice, the expression of CCR6 and CCL20 is increased in the kidney with lupus development [24]. Renal expression of CCL20 is also increased in diseased MRL/lpr mice [23]. These results suggest that CCR6 and CCL20 may function to regulate LN by recruiting Th17 and Treg cells.

## 3. Mechanisms of Chemokine Induction in Lupus Nephritic Kidney

As summarized above, in the kidney of both SLE patients and lupus-prone mice, many chemokines rarely expressed at steady state are induced or significantly increased with LN progression, suggesting that local and/or systemic inflammatory factors may trigger the upregulation of these chemokines. As summarized in Table 3, targeting both renal parenchymal cells and renal-infiltrating immune cells, nucleic acid-containing antigens and autoantibodies are considered to be the major inflammatory stimulators initiating and/or accelerating chemokine release in the lupus nephritic kidney.

Mesangial cells and other intrinsic renal cells like glomerular capillary endothelial and proximal tubular epithelial cells that express several toll-like receptors (TLRs) have the potential to be activated by different antigens to produce inflammatory factors including chemokines. PolyI:C RNA that mimics viral dsRNA can induce mesangial cells from MRL/lpr mice to produce CCL2, whereas mesangial cells from humans can be activated by polyI:C to produce CXCL1 through the TLR3 signaling pathway [112, 113]. Mesangial cells from lupus-prone mice, compared to nonlupus mice, were more sensitive to lipopolysaccharide (LPS) stimulation as shown by the higher TLR4, MyD88, and NF*κ*B expression and higher CCL2 production, suggesting a mechanism of how bacterial infections accelerate lupus disease [114].

Besides exogenous factors, primary mesangial cells isolated from NZB/W F1 mice, upon self-nucleosome or nucleosome-containing IC stimulation* in vitro*, have been shown to produce several chemokines including CCL2, CCL7, CCL20, CXCL2, and CXCL5, suggesting self-antigen and autoantibody-mediated mesangial activation [115]. Regarding autoantibody-induced mesangial activation, it has been shown that pathogenic anti-dsDNA IgG can upregulate CXCL1 and CX3CL1 transcripts and the secretion of CXCL1 from mesangial cells isolated from MRL/lpr mice through both Fc receptor- (FcR- ) dependent and independent pathways [96]. Further studies have shown that FcR-independent pathway is dependent on TLR2/4 and the Receptor for Advanced Glycation End products (RAGE) but independent of DNA/TLR9, as pathogenic anti-dsDNA IgG clone 1A3F can bind high mobility group box 1, an endogenous ligand for TLR2/4 and RAGE, through which 1A3F activates TLR2/RAGE-MyD88-NF*κ*B pathway in mesangial cells, leading to the production of several chemokines including CXCL1, CXCL2, CXCL5, CXCL16, CCL7, and CCL20 [96, 116]. Autoantibodies can also indirectly activate intrinsic renal cells. When incubated with immobilized IgG mimicking IC deposition in the kidney, lymphocytes isolated from human PBMC can be activated in a Fc*γ*R-dependent way to produce IL-1*β* that in turn stimulates human mesangial cells, glomerular capillary endothelial cells, and proximal tubular epithelial cells to further produce CCL2 [117]. Moreover, a transcription factor, Fli1, has been shown to directly bind the promoter region of CCL2 and CCL5 genes to promote their expression in primary endothelial cells of the kidney in NZM2410 lupus-prone mice [118, 119].

In addition to the activation of renal parenchymal cells, renal-infiltrating macrophages and dendritic cells have been shown to produce several chemokines upon stimulation by TLR2/4, TLR3, TLR7, and TLR9 ligands. Biglycan, an endogenous stimulator of TLR2/4, is increased in the serum and kidney of both SLE patients and MRL/lpr mice [120]. An* in vitro* study further shows that macrophages and dendritic cells produce CXCL13 upon biglycan activation of TLR2/4-ROS signaling pathway that is independent of inflammasome. PolyI:C RNA mimicking viral dsRNA and a TLR7 agonist mimicking viral ssRNA can induce macrophages and dendritic cells isolated from MRL/lpr mice to produce CCL2 through the TLR3 and TLR7 signaling pathways, respectively [112, 121]. In addition, it has been shown that TLR7 and TLR9 are mostly detected in renal-infiltrating macrophages and dendritic cells rather than intrinsic renal cells of MRL/lpr mice [26, 121]. CpG, mimicking bacterial DNA or self-dsDNA, can induce CCL2, CCL5, and CCR5 in the kidney through the TLR9 pathway when injected into MRL/lpr mice [26]. Detailed studies have shown that exogenous CpG or bacterial DNA particularly bind to infiltrating macrophages and dendritic cells in the glomerular and tubulointerstitial regions of the kidney in MRL/lpr mice [26]. Chloroquine-blocked TLR9 pathway abolishes CCL5 induction in spleen monocytes, further demonstrating TLR9-dependent chemokine induction in renal-infiltrating innate immune cells upon CpG-DNA triggering [26]. Finally, miRNA-125a has been shown to indirectly downregulate CCL5 production by activated T cells through targeting Kruppel-like factor 13 (KLF13) [122]. It has been demonstrated that miRNA-125a is downregulated while KLF13/CCL5 are upregulated in PBMC of SLE patients compared to healthy controls, suggesting that dysregulation of CCL5 in SLE patients is dependent on miRNA-125a.

## 4. Chemokines as Biomarkers for LN

To date, renal biopsy is still the gold standard for accurate diagnosis and classification of LN and for the prognosis of LN activity and chronicity in patients upon treatment. However, chemokines in the urine of lupus nephritic patients have been studied according to established diagnosis of LN classes that suggest their potential use as noninvasive biomarkers for LN activity monitoring, treatment responses, and remission/flare prediction after the biopsy diagnosis.

Urine chemokines can be used to supplement renal biopsy diagnosis of LN. In both adult and juvenile SLE patients, urinary CCL2 (uCCL2) concentration is significantly higher in nephritic patients than nonnephritic patients and healthy controls [123, 124]. Moreover, both protein and mRNA levels of uCCL2 are significantly higher in SLE patients with active LN compared to those with inactive LN [77, 125–130]. Further studies have shown that uCCL2 alone or combined with other factors can distinguish different classes of LN, as uCCL2 concentration is positively correlated to progressive LN classes and significantly increased in diffuse proliferative group compared to focal proliferative and mesangioproliferative groups [123, 131, 132]. In addition, as interstitial lesions are always associated with end-stage LN, it is important to monitor uCCL2 whose level is high during interstitial inflammation in moderate-severe SLE patients [133]. Besides uCCL2, urinary CXCL10 (uCXCL10) concentration is also significantly higher in nephritic patients than nonnephritic SLE patients [134]. A cut-off value 93 pg/dL of uCXCL10 has been proved to be a good prediction of nephritis with high sensitivity and specificity. Also, uCXCL10 concentration is positively correlated with renal activity score and renal biopsy grade. In addition, uCXCL10 and CXCR3 mRNA levels from class IV nephritic patients are increased compared to other classes [135]. Similarly, the urinary CXCL16 level can also distinguish inactive and active LN in SLE patients [114].

During the treatment of LN, urinary chemokines can be useful for monitoring treatment responses. uCCL2 has been shown to be a good biomarker to predict juvenile LN improvement [136]. In this study, the cut-off uCCL2 concentration is 343 pg/mL, with a value lower than that predicting an improved renal disease activity. Two other studies have also shown that uCCL2 is reduced in SLE patients with complete or partial LN remission, while its level is maintained in nonremission patients, suggesting uCCL2 as a good marker for prognosis [129, 132]. Similarly, uCXCL10 is reduced in SLE patients upon remission into inactive LN in a longitudinal follow-up study, suggesting uCXCL10 is also a good biomarker for monitoring LN improvement of SLE patients following the treatment [137]. After the remission, the elevation of uCCL2 can be detected 2 to 4 months prior to another LN flare, and changes of uCCL2 concentration can distinguish different levels of LN flare severity, suggesting that uCCL2 may be a good marker for predicting recurring LN flares [129, 132].

## 5. Potential Role of Chemokine-Based Drugs to Treat LN

As chemokines and chemokine receptors are important in the recruitment of leukocytes to the kidney in the development of LN, one would naturally think of developing new treatments for LN that target the interaction between chemokines and chemokine receptors. However, the design of such treatments should take into consideration the potential limitations (discussed below), as many commercial drugs designed to target chemokines/chemokine receptors in different diseases have unfortunately failed in clinical trials (summarized tables in references) [138, 139].

The complexity of chemokine and chemokine receptor system and possible redundancy are a challenge for the development of new drugs to block leukocyte infiltration [139, 140]. Some chemokines, such as CCL5, can recognize several chemokine receptors (CCR1, CCR3, and CCR5), whereas some chemokine receptors, such as CXCR3, can interact with different chemokines (CXCL9, CXCL10, and CXCL11) [139]. Current drugs including small chemical molecules and monoclonal antibodies are designed to simply block the interactions between chemokines and chemokine receptors by neutralizing either chemokines or chemokine receptors, which is insufficient to pinpoint the specific function of each chemokine/chemokine receptor pair. Thus, detailed studies on the dynamic interactions and functions of each particular chemokine/chemokine receptor pair in the specific diseases are critical for successful drug development. In addition, the chemokine and chemokine receptor redundancy is reflected in a situation where one chemokine receptor may function in compensation of another if the other chemokine receptor is blocked. Leukocytes always express more than one type of chemokine receptors on the surface, so blocking the ligation of one chemokine receptor may not completely or efficiently prevent the infiltration of leukocytes. For example, while both CCR5 and CXCR3 have been shown to promote organ transplantation rejection by inducing T cells infiltration in the transplanted organ, their functions seem to be redundant. CCR5 and CXCR3 double blocking compared to either single blocking makes a much greater prolonged allograft survival in a murine heterotopic heart transplantation model [141]. Hence, future studies are necessary to clarify the effect of chemokine receptor compensation.

Another challenge for LN drug development that involves chemokines and chemokine receptors is to achieve cell-specific targeting. To this end, studies of chemokine receptor expression at the single cell level may help identify the cell type of interest. For example, renal-infiltrating T cells have been shown to express CCR1, CCR4, CCR5, CXCR3, and CXCR5 [24, 50, 67, 70, 87]. However, it is unknown whether the chemokine receptors are expressed on the same T cell subsets or differentially expressed on distinct T cell subsets. If we can define T cell subsets by using different combinations of chemokine receptors, we may be able to more specifically target pathogenic T cells by blocking the corresponding chemokine receptors.

An additional limitation is the use of lupus-prone mice where most of the mechanistic studies are performed to better understand the pathogenesis of LN. The differences between human patients and mouse models make it difficult to translate the results of mouse studies to successful clinical trials. Therefore, it is important to study the differences and similarities between SLE patients and lupus-prone mice regarding their use of chemokines and chemokine receptors. An example is CXCL9, which is preferentially used in mice but not in humans, versus CXCL10, which appears to be the predominant chemokine in the kidney of SLE patients [23, 67].

Finally, how to specifically deliver chemokine-based drugs into the kidney is another important question. It is difficult to find a kidney-specific chemokine or chemokine receptor critical for the development of LN. Systemically blocking a chemokine or chemokine receptor will likely lead to many outcomes other than attenuating LN, causing negative side effects such as an increased chance of infection or cancer. Therefore, while targeting the interaction between chemokines and chemokine receptors is a promising avenue, further studies are required to dissect and better understand the mechanisms behind such interactions before a chemokine-based drug can be developed to treat LN.

Although many commercial chemokine receptor antagonists failed to reach expectations in treating different diseases, targeting chemokines/chemokine receptors may still be a promising strategy in LN. First, studies using SLE patient cells/tissue and animal models summarized in this review have demonstrated the involvement of chemokines/chemokine receptors in LN progression, suggesting the potential of targeting this system in LN treatment. Second, the failure of previously designed drugs is due to our insufficient understanding of the complicated chemokine/chemokine receptor system, which can be improved by further studies. Third, with better understanding of chemokine/chemokine receptor system, future drugs designed to more specifically target particular chemokine and chemokine receptor interactions will minimize the off-target effects and side effects commonly observed for immunosuppressive drugs and monoclonal antibodies, which are nonspecific. Finally, we may be able to learn from pathogens that are known to specifically target chemokine/chemokine receptor pairs [139] to design better drugs with improved specificity.

## Figures and Tables

**Table 1:** Target cells and roles of chemokine receptors and chemokines in lupus-prone mouse models of LN.

Chemokine receptor	Chemokine(s)	Roles	Reference(s)
CXCR5	CXCL13	*Targets*: B cells, B1 cells, T_FH_ *Role*: promote both systemic response and local renal inflammation	[24, 26, 30–32, 34]

CXCR4	CXCL12	*Targets*: B cells, CD4^+^Foxp3^+^ T cells, CD4^+^Foxp3^−^ T cells, plasma cells, neutrophils, monocytes, PEC *Role*: promote both systemic response and local renal inflammation	[39–42, 44]

CXCR3	CXCL9	*Targets*: CD4^+^ T cells, CD8^+^ T cells, B220^+^ cells, plasma cells, macrophages *Role*: promote local renal inflammation in MRL/lpr mice	[23, 48, 49]

CCR1	CCL3, 5	*Targets*: T cells, monocytes/macrophages *Role*: promote local renal inflammation	[26, 50, 51]

CCR5	CCL3, 5	*Targets*: T cells, Foxp3^+^ Treg cells, macrophages *Role*: negatively regulate systemic response and local renal inflammation	[51–53]

CCR2	CCL2	*Target*: macrophages *Role*: promote both systemic response and local renal inflammation	[52, 54–59]

CX3CR1	CX3CL1	*Target*: CD16^+^ cells *Role*: promote local renal inflammation	[60, 61]

**Table 2:** Chemokine receptors and chemokines likely to mediate cell infiltration into the kidney in human LN.

Chemokine receptor	Chemokine(s)	Target cell type(s)	Reference(s)
CXCR5	CXCL13	Podocytes, B cells, T_FH_	[34, 35]
CXCR4	CXCL12	B cells, CD4^+^ T cells	[62–65]
CXCR3	CXCL10	CD4^+^ T cells, T cells, CD19^high^ B cells	[26, 63, 66–68]
CCR1	CCL3, 5	CD68^+^ macrophages	[63, 69]
CCR5	CCL3, 4, 5	T cells	[70]
CCR2	CCL2	CD4^+^ T cells, CD8^+^ T cells	[63]
CX3CR1	CX3CL1	CD16^+^ cells	[71]

**Table 3:** Mechanisms of chemokine induction in lupus nephritic kidney.

Stimulators	Target cells	Signaling pathways	Chemokines
Self-nucleosome or nucleosome-containing IC	Mesangial cells	FcR, TLR2/4-RAGE, MyD88, NF*κ*B	CCL2, CCL7, CCL20, CXCL2, CXCL5

Pathogenic anti-dsDNA IgG	Mesangial cells	FcR, TLR2/4-RAGE, MyD88, NF*κ*B	CXCL1, CXCL2, CXCL5, CXCL16, CCL7, CCL20, CX3CL1

Lymphocyte activated by immobilized IgG	Mesangial cells, glomerular capillary endothelial, proximal tubular epithelial cells	IL-1*β*	CCL2

LPS	Mesangial cells	TLR4, MyD88, NF*κ*B	CCL2

LPS	Glomerular capillary endothelial	Fli1 transcription factor	CCL2, CCL5

Poly I:C RNA	Mesangial cells	TLR3, IFN*β*, IRF3, NF*κ*B	CCL2, CXCL1

Poly I:C RNA	Infiltrating macrophages and DC	TLR3	CCL2

RNA40, imiquimod	Infiltrating macrophages and DC	TLR7	CCL2

CpG-ODN, bacterial DNA	Infiltrating macrophages and DC	TLR9	CCL2, CCL5

Biglycan	Infiltrating macrophages and DC	TLR2/4, ROS	CXCL13

Phytohemagglutinin	T cells	miRNA-125a, KLF13	CCL5

## Additional Points

Chemokines and chemokine receptors contribute to LN development by involvement in both autoimmune initiation in immune tissues and amplification of inflammation in the nephritic kidney. Various leukocyte populations migrate into the lupus nephritic kidney through the interactions of chemokines and chemokine receptors, which, together with inflammatory chemokine-activated renal parenchymal cells, lead to acute and chronic LN. Further studies of LN should be more focused on cell-specific chemokine/chemokine receptor functions through the development of cell-specific knockouts. Moreover, detailed studies of particular chemokine/chemokine receptor interactions, as well as studies of the similarities and differences between mouse models and human patients, will serve as the basis for future drug development that benefit SLE patients with LN.

## References

[1] Tsokos G. C. (2011). Mechanisms of disease: Systemic lupus erythematosus. *The New England Journal of Medicine*.

[2] Saxena R., Mahajan T., Mohan C. (2011). Lupus nephritis: current update. *Arthritis Research & Therapy*.

[3] Lech M., Anders H.-J. (2013). The pathogenesis of lupus nephritis. *Journal of the American Society of Nephrology*.

[4] Ransohoff R. M. (2009). Chemokines and chemokine receptors: standing at the crossroads of immunobiology and neurobiology. *Immunity*.

[5] Griffith J. W., Sokol C. L., Luster A. D. (2014). Chemokines and chemokine receptors: positioning cells for host defense and immunity. *Annual Review of Immunology*.

[6] Chung A. C. K., Lan H. Y. (2011). Chemokines in renal injury. *Journal of the American Society of Nephrology*.

[7] Panzer U., Steinmetz O. M., Stahl R. A. K., Wolf G. (2006). Kidney diseases and chemokines. *Current Drug Targets*.

[8] Charo I. F., Ransohoff R. M. (2006). Mechanisms of disease: the many roles of chemokines and chemokine receptors in inflammation. *The New England Journal of Medicine*.

[9] Zlotnik A., Burkhardt A. M., Homey B. (2011). Homeostatic chemokine receptors and organ-specific metastasis. *Nature Reviews Immunology*.

[10] Nowling T. K., Gilkeson G. S. (2011). Mechanisms of tissue injury in lupus nephritis. *Arthritis Research and Therapy*.

[11] Spada R., Rojas J. M., Pérez-Yagüe S. (2015). NKG2D ligand overexpression in lupus nephritis correlates with increased NK cell activity and differentiation in kidneys but not in the periphery. *Journal of Leukocyte Biology*.

[12] Förster R., Mattis A. E., Kremmer E., Wolf E., Brem G., Lipp M. (1996). A putative chemokine receptor, BLR1, directs B cell migration to defined lymphoid organs and specific anatomic compartments of the spleen. *Cell*.

[13] Quigley M. F., Gonzalez V. D., Granath A., Andersson J., Sandberg J. K. (2007). CXCR5^+^ CCR7- CD8 T cells are early effector memory cells that infiltrate tonsil B cell follicles. *European Journal of Immunology*.

[14] León B., Ballesteros-Tato A., Browning J. L., Dunn R., Randall T. D., Lund F. E. (2012). Regulation of T H2 development by CXCR5^+^ dendritic cells and lymphotoxin-expressing B cells. *Nature Immunology*.

[15] Schaerli P., Willimann K., Lang A. B., Lipp M., Loetscher P., Moser B. (2000). CXC chemokine receptor 5 expression defines follicular homing T cells with B cell helper function. *The Journal of Experimental Medicine*.

[16] Kim C. H., Kunkel E. J., Boisvert J. (2001). Bonzo/CXCR6 expression defines type 1-polarized T-cell subsets with extralymphoid tissue homing potential. *The Journal of Clinical Investigation*.

[17] Crotty S. (2011). Follicular helper CD4 T cells (TFH). *Annual Review of Immunology*.

[18] Chevalier N., Jarrossay D., Ho E. (2011). CXCR5 expressing human central memory CD4 T cells and their relevance for humoral immune responses. *The Journal of Immunology*.

[19] Locci M., Havenar-Daughton C., Landais E. (2013). Human circulating PD-1^+^CXCR3^−^CXCR5^+^ memory Tfh cells are highly functional and correlate with broadly neutralizing HIV antibody responses. *Immunity*.

[20] Bentebibel S.-E., Lopez S., Obermoser G. (2013). Induction of ICOS^+^CXCR3^+^CXCR5^+^T_H_ cells correlates with antibody responses to influenza vaccination. *Science Translational Medicine*.

[21] Slight S. R., Rangel-Moreno J., Gopal R. (2013). CXCR5^+^ T helper cells mediate protective immunity against tuberculosis. *The Journal of Clinical Investigation*.

[22] Ishikawa S., Sato T., Abe M. (2001). Aberrant high expression of B lymphocyte chemokine (BLC/CXCL13) by C11b^+^CD11c^+^ dendritic cells in murine lupus and preferential chemotaxis of B1 cells towards BLC. *The Journal of Experimental Medicine*.

[23] Menke J., Zeller G. C., Kikawada E. (2008). CXCL9, but not CXCL10, promotes CXCRS-dependent immune-mediated kidney disease. *Journal of the American Society of Nephrology*.

[24] Schiffer L., Bethunaickan R., Ramanujam M. (2008). Activated renal macrophages are markers of disease onset and disease remission in lupus nephritis. *Journal of Immunology*.

[25] Adalid-Peralta L., Mathian A., Tran T. (2008). Leukocytes and the kidney contribute to interstitial inflammation in lupus nephritis. *Kidney International*.

[26] Anders H.-J., Belemezova E., Eis V. (2004). Late onset of treatment with a chemokine receptor CCR1 antagonist prevents progression of lupus nephritis in MRL-Fas(lpr) mice. *Journal of the American Society of Nephrology*.

[27] Schiffer L., Worthmann K., Haller H., Schiffer M. (2015). CXCL13 as a new biomarker of systemic lupus erythematosus and lupus nephritis—from bench to bedside?. *Clinical and Experimental Immunology*.

[28] Clark K. L., Reed T. J., Wolf S. J., Lowe L., Hodgin J. B., Kahlenberg J. M. (2015). Epidermal injury promotes nephritis flare in lupus-prone mice. *Journal of Autoimmunity*.

[29] Ishikawa S., Nagai S., Sato T. (2002). Increased circulating CD11b^+^CD11c^+^ dendritic cells (DC) in aged BWF1 mice which can be matured by TNF-*α* into BLC/CXCL13-producing DC. *European Journal of Immunology*.

[30] Ishikawa S., Matsushima K. (2007). Aberrant B1 cell trafficking in a murine model for lupus. *Frontiers in Bioscience*.

[31] Doerner J. L., Wen J., Xia Y. (2015). TWEAK/Fn14 signaling involvement in the pathogenesis of cutaneous disease in the MRL/lpr model of spontaneous lupus. *Journal of Investigative Dermatology*.

[32] Wiener A., Schippers A., Wagner N. (2016). CXCR5 deficiency ameliorates murine lupus. *Clinical & Experimental Immunology*.

[33] Espeli M., Bökers S., Giannico G. (2011). Local renal autoantibody production in lupus nephritis. *Journal of the American Society of Nephrology*.

[34] Steinmetz O. M., Velden J., Kneissler U. (2008). Analysis and classification of B-cell infiltrates in lupus and ANCA-associated nephritis. *Kidney International*.

[35] Worthmann K., Gueler F., Von Vietinghoff S. (2014). Pathogenetic role of glomerular CXCL13 expression in lupus nephritis. *Clinical and Experimental Immunology*.

[36] Guyon A. (2014). CXCL12 chemokine and its receptors as major players in the interactions between immune and nervous systems. *Frontiers in Cellular Neuroscience*.

[37] Daridon C., Blassfeld D., Reiter K. (2010). Epratuzumab targeting of CD22 affects adhesion molecule expression and migration of B-cells in systemic lupus erythematosus. *Arthritis Research & Therapy*.

[38] Klein R. S., Rubin J. B. (2004). Immune and nervous system CXCL12 and CXCR4: parallel roles in patterning and plasticity. *Trends in Immunology*.

[39] Badr G., Sayed A., Abdel-Maksoud M. A. (2015). Infection of female BWF1 lupus mice with malaria parasite attenuates B cell autoreactivity by modulating the CXCL12/CXCR4 axis and its downstream signals PI3K/AKT, NF*κ*B and ERK. *PLoS ONE*.

[40] Jin O., Zhang H., Gu Z. (2009). A pilot study of the therapeutic efficacy and mechanism of artesunate in the MRL/lpr murine model of systemic lupus erythematosus. *Cellular & Molecular Immunology*.

[41] Abe J., Ueha S., Suzuki J., Tokano Y., Matsushima K., Ishikawa S. (2008). Increased Foxp3^+^ CD4^+^ regulatory T cells with intact suppressive activity but altered cellular localization in murine lupus. *American Journal of Pathology*.

[42] Balabanian K., Couderc J., Bouchet-Delbos L. (2003). Role of the chemokine stromal cell-derived factor 1 in autoantibody production and nephritis in murine lupus. *Journal of Immunology*.

[43] Feng X., Che N., Liu Y. (2014). Restored immunosuppressive effect of mesenchymal stem cells on b cells after olfactory 1/early B cell factor-associated zinc-finger protein down-regulation in patients with systemic lupus erythematosus. *Arthritis & Rheumatology*.

[44] Rizzo P., Perico N., Gagliardini E. (2013). Nature and mediators of parietal epithelial cell activation in glomerulonephritides of human and rat. *The American Journal of Pathology*.

[45] Mazzinghi B., Ronconi E., Lazzeri E. (2008). Essential but differential role for CXCR4 and CXCR7 in the therapeutic homing of human renal progenitor cells. *The Journal of Experimental Medicine*.

[46] Shankland S. J., Anders H.-J., Romagnani P. (2013). Glomerular parietal epithelial cells in kidney physiology, pathology, and repair. *Current Opinion in Nephrology and Hypertension*.

[47] Trivedi S., Zeier M., Reiser J. (2009). Role of podocytes in lupus nephritis. *Nephrology Dialysis Transplantation*.

[48] Moser K., Kalies K., Szyska M., Humrich J. Y., Amann K., Manz R. A. (2012). CXCR3 promotes the production of IgG1 autoantibodies but is not essential for the development of lupus nephritis in NZB/NZW mice. *Arthritis and Rheumatism*.

[49] Odegard J. M., DiPlacido L. D., Greenwald L. (2009). ICOS controls effector function but not trafficking receptor expression of kidney-infiltrating effector T cells in murine lupus. *Journal of Immunology*.

[50] Bignon A., Gaudin F., Hémon P. (2014). CCR1 inhibition ameliorates the progression of lupus nephritis in NZB/W mice. *Journal of Immunology*.

[51] Moore K. J., Wada T., Barbee S. D., Kelley V. R. (1998). Gene transfer of RANTES elicits autoimmune renal injury in MRL-**F**
**a**
**s**
^lpr^ mice. *Kidney International*.

[52] Vielhauer V., Anders H.-J., Pérez de Lema G., Luckow B., Schlöndorff D., Mack M. (2003). Phenotyping renal leukocyte subsets by four-color flow cytometry: characterization of chemokine receptor expression. *Nephron. Experimental Nephrology*.

[53] Turner J.-E., Paust H.-J., Bennstein S. B. (2012). Protective role for CCR5 in murine lupus nephritis. *American Journal of Physiology—Renal Physiology*.

[54] Pérez de Lema G., Maier H., Nieto E. (2001). Chemokine expression precedes inflammatory cell infiltration and chemokine receptor and cytokine expression during the initiation of murine lupus nephritis. *Journal of the American Society of Nephrology*.

[55] Tesch G. H., Maifert S., Schwarting A., Rollins B. J., Kelley V. R. (1999). Monocyte chemoattractant protein 1-dependent leukocytic infiltrates are responsible for autoimmune disease in MRL-**F**
**a**
**s**
^lpr^ mice. *The Journal of Experimental Medicine*.

[56] Hasegawa H., Kohno M., Sasaki M. (2003). Antagonist of monocyte chemoattractant protein 1 ameliorates the initiation and progression of lupus nephritis and renal vasculitis in MRL/lpr mice. *Arthritis and Rheumatism*.

[57] De Lema G. P., Maier H., Franz T. J. (2005). Chemokine receptor Ccr2 deficiency reduces renal disease and prolongs survival in MRL/lpr lupus-prone mice. *Journal of the American Society of Nephrology*.

[58] Nakashima H., Akahoshi M., Shimizu S. (2004). Absence of association between the MCP-1 gene polymorphism and histological phenotype of lupus nephritis. *Lupus*.

[59] Kulkarni O., Pawar R. D., Purschke W. (2007). Spiegelmer inhibition of CCL2/MCP-1 ameliorates lupus nephritis in MRL-(Fas)lpr mice. *Journal of the American Society of Nephrology*.

[60] Nakatani K., Yoshimoto S., Iwano M. (2010). Fractalkine expression and CD16^+^ monocyte accumulation in glomerular lesions: association with their severity and diversity in lupus models. *American Journal of Physiology—Renal Physiology*.

[61] Inoue A., Hasegawa H., Kohno M. (2005). Antagonist of fractalkine (CX3CL1) delays the initiation and ameliorates the progression of lupus nephritis in MRL/lpr mice. *Arthritis and Rheumatism*.

[62] Wang A., Guilpain P., Chong B. F. (2010). Dysregulated expression of CXCR4/CXCL12 in subsets of patients with systemic lupus erythematosus. *Arthritis and Rheumatism*.

[63] Amoura Z., Combadiere C., Faure S. (2003). Roles of CCR2 and CXCR3 in the T cell-mediated response occurring during lupus flares. *Arthritis and Rheumatism*.

[64] Biajoux V., Bignon A., Freitas C. (2012). Expression of CXCL12 receptors in B cells from Mexican mestizos patients with systemic lupus erythematosus. *Journal of Translational Medicine*.

[65] Hanaoka H., Okazaki Y., Hashiguchi A. (2015). Overexpression of CXCR4 on circulating B cells in patients with active systemic lupus erythematosus. *Clinical and Experimental Rheumatology*.

[66] Steinmetz O. M., Turner J.-E., Paust H.-J. (2009). CXCR3 mediates renal Th1 and Th17 immune response in murine lupus nephritis. *The Journal of Immunology*.

[67] Enghard P., Humrich J. Y., Rudolph B. (2009). CXCR3+CD4+ T cells are enriched in inflamed kidneys and urine and provide a new biomarker for acute nephritis flares in systemic lupus erythematosus patients. *Arthritis and Rheumatism*.

[68] Nicholas M. W., Dooley M. A., Hogan S. L. (2008). A novel subset of memory B cells is enriched in autoreactivity and correlates with adverse outcomes in SLE. *Clinical Immunology*.

[69] Furuichi K., Wada T., Sakai N. (2000). Distinct expression of CCR1 and CCR5 in glomerular and interstitial lesions of human glomerular diseases. *American Journal of Nephrology*.

[70] Segerer S., Mack M., Regele H., Kerjaschki D., Schlöndorff D. (1999). Expression of the C–C chemokine receptor 5 in human kidney diseases. *Kidney International*.

[71] Yoshimoto S., Nakatani K., Iwano M. (2007). Elevated levels of fractalkine expression and accumulation of CD16+ monocytes in glomeruli of active lupus nephritis. *American Journal of Kidney Diseases*.

[72] Groom J. R., Luster A. D. (2011). CXCR3 ligands: redundant, collaborative and antagonistic functions. *Immunology and Cell Biology*.

[73] Lacotte S., Brun S., Muller S., Dumortier H. (2009). CXCR3, inflammation, and autoimmune diseases. *Annals of the New York Academy of Sciences*.

[74] Lacotte S., Decossas M., Le Coz C., Brun S., Muller S., Dumortier H. (2013). Early differentiated CD138^high^MHCII^+^IgG^+^ plasma cells express CXCR3 and localize into inflamed kidneys of lupus mice. *PLoS ONE*.

[75] Teramoto K., Negoro N., Kitamoto K. (2008). Microarray analysis of glomerular gene expression in murine lupus nephritis. *Journal of Pharmacological Sciences*.

[76] Pestka J. J., Vines L. L., Bates M. A., He K., Langohr I. (2014). Comparative effects of n-3, n-6 and n-9 unsaturated fatty acid-rich diet consumption on lupus nephritis, autoantibody production and CD4^+^ T cell-related gene responses in the autoimmune NZBWF1 mouse. *PLoS ONE*.

[77] Segawa C., Wada T., Furuichi K., Takasawa K., Yokoyama H., Kobayashi K.-I. (1996). Steroid pulse therapy in lupus cystitis. *Internal Medicine*.

[78] Reichel C. A., Khandoga A., Anders H.-J., Schlöndorff D., Luckow B., Krombach F. (2006). Chemokine receptors Ccr1, Ccr2, and Ccr5 mediate neutrophil migration to postischemic tissue. *Journal of Leukocyte Biology*.

[79] Mackay C. R. (2014). CXCR3^+^CCR5^+^T cells and autoimmune diseases: guilty as charged?. *The Journal of Clinical Investigation*.

[80] Weber C., Weber K. S. C., Klier C. (2001). Specialized roles of the chemokine receptors CCR1 and CCR5 in the recruitment of monocytes and TH1-like/CD45RO+ T cells. *Blood*.

[81] Eis V., Luckow B., Vielhauer V. (2004). Chemokine receptor CCR1 but not CCR5 mediates leukocyte recruitment and subsequent renal fibrosis after unilateral ureteral obstruction. *Journal of the American Society of Nephrology*.

[82] Anders H.-J., Vielhauer V., Frink M. (2002). A chemokine receptor CCR-1 antagonist reduces renal fibrosis after unilateral ureter ligation. *The Journal of Clinical Investigation*.

[83] Furuichi K., Gao J.-L., Horuk R., Wada T., Kaneko S., Murphy P. M. (2008). Chemokine receptor CCR1 regulates inflammatory cell infiltration after renal ischemia-reperfusion injury. *Journal of Immunology*.

[84] Topham P. S., Csizmadia V., Soler D. (1999). Lack of chemokine receptor CCR1 enhances Th1 responses and glomerular injury during nephrotoxic nephritis. *The Journal of Clinical Investigation*.

[85] Gao W., Topham P. S., King J. A. (2000). Targeting of the chemokine receptor CCR1 suppresses development of acute and chronic cardiac allograft rejection. *The Journal of Clinical Investigation*.

[86] Stasikowska O., Danilewicz M., Wagrowska-Danilewicz M. (2007). The significant role of RANTES and CCR5 in progressive tubulointerstitial lesions in lupus nephropathy. *Polish Journal of Pathology*.

[87] Yamada M., Yagita H., Inoue H. (2002). Selective accumulation of CCR4+ T lymphocytes into renal tissue of patients with lupus nephritis. *Arthritis and Rheumatism*.

[88] Deshmane S. L., Kremlev S., Amini S., Sawaya B. E. (2009). Monocyte chemoattractant protein-1 (MCP-1): an overview. *Journal of Interferon and Cytokine Research*.

[89] Zoja C., Liu X.-H., Donadelli R. (1997). Renal expression of monocyte chemoattractant protein-1 in lupus autoimmune mice. *Journal of the American Society of Nephrology*.

[90] Bazan J. F., Bacon K. B., Hardiman G. (1997). A new class of membrane-bound chemokine with a CX3C motif. *Nature*.

[91] Imai T., Hieshima K., Haskell C. (1997). Identification and molecular characterization of fractalkine receptor CX3CR1, which mediates both leukocyte migration and adhesion. *Cell*.

[92] Soos T. J., Sims T. N., Barisoni L. (2006). CX3CR1+ interstitial dendritic cells form a contiguous network throughout the entire kidney. *Kidney International*.

[93] Niess J. H., Adler G. (2010). Enteric flora expands gut lamina propria CX3CR1^+^ dendritic cells supporting inflammatory immune responses under normal and inflammatory conditions. *Journal of Immunology*.

[94] Landsman L., Liat B.-O., Zernecke A. (2009). CX_3_CR1 is required for monocyte homeostasis and atherogenesis by promoting cell survival. *Blood*.

[95] Lionakis M. S., Swamydas M., Fischer B. G. (2013). CX3CR1-dependent renal macrophage survival promotes Candida control and host survival. *Journal of Clinical Investigation*.

[96] Qing X., Zavadil J., Crosby M. B. (2006). Nephritogenic anti-DNA antibodies regulate gene expression in MRL/lpr mouse glomerular mesangial cells. *Arthritis and Rheumatism*.

[97] Imai T., Nagira M., Takagi S. (1999). Selective recruitment of CCR4-bearing T_h_2 cells toward antigen-presenting cells by the CC chemokines thymus and activation-regulated chemokine and macrophage-derived chemokine. *International Immunology*.

[98] Turner J.-E., Paust H.-J., Steinmetz O. M. (2010). CCR6 recruits regulatory T cells and Th17 cells to the kidney in glomerulonephritis. *Journal of the American Society of Nephrology*.

[99] Nanki T., Shimaoka T., Hayashida K., Taniguchi K., Yonehara S., Miyasaka N. (2005). Pathogenic role of the CXCL16-CXCR6 pathway in rheumatoid arthritis. *Arthritis and Rheumatism*.

[100] Garcia G. E., Truong L. D., Li P. (2007). Inhibition of CXCL16 attenuates inflammatory and progressive phases of anti-glomerular basement membrane antibody-associated glomerulonephritis. *The American Journal of Pathology*.

[101] Iikuni N., Okamoto H., Yoshio T. (2006). Raised monocyte chemotactic protein-1 (MCP-1)/CCL2 in cerebrospinal fluid of patients with neuropsychiatric lupus. *Annals of the Rheumatic Diseases*.

[102] Qin M., Guo Y., Jiang L., Wang X. (2014). Elevated levels of serum sCXCL16 in systemic lupus erythematosus; potential involvement in cutaneous and renal manifestations. *Clinical Rheumatology*.

[103] Li G.-Y., Lu X.-Q., Liu S.-X. (2011). Langohuangping granule down-regulated monocyte chemoattractant protein-1 via suppressing nuclear factor kappa B signaling pathway in BXSB lupus nephritis mice. *Zhongguo Zhong Xi Yi Jie He Za Zhi Zhongguo Zhongxiyi Jiehe Zazhi*.

[104] Yamazaki T., Yang X. O., Chung Y. (2008). CCR6 regulates the migration of inflammatory and regulatory T cells. *Journal of Immunology*.

[105] Rivino L., Gruarin P., Häringer B. (2010). CCR6 is expressed on an IL-10-producing, autoreactive memory T cell population with context-dependent regulatory function. *Journal of Experimental Medicine*.

[106] Wiede F., Fromm P. D., Comerford I. (2013). CCR6 is transiently upregulated on B cells after activation and modulates the germinal center reaction in the mouse. *Immunology and Cell Biology*.

[107] Cook D. N., Prosser D. M., Forster R. (2000). CCR6 mediates dendritic cell localization, lymphocyte homeostasis, and immune responses in mucosal tissue. *Immunity*.

[108] Salazar-Gonzalez R. M., Niess J. H., Zammit D. J. (2006). CCR6-mediated dendritic cell activation of pathogen-specific T cells in Peyer's patches. *Immunity*.

[109] Kucharzik T., Hudson J. T., Waikel R. L., Martin W. D., Williams I. R. (2002). CCR6 expression distinguishes mouse myeloid and lymphoid dendritic cell subsets: demonstration using a CCR6 EGFP knock-in mouse. *European Journal of Immunology*.

[110] Kluger M. A., Melderis S., Nosko A. (2016). Treg17 cells are programmed by Stat3 to suppress Th17 responses in systemic lupus. *Kidney International*.

[111] Hirota K., Yoshitomi H., Hashimoto M. (2007). Preferential recruitment of CCR6-expressing Th17 cells to inflamed joints via CCL20 in rheumatoid arthritis and its animal model. *The Journal of Experimental Medicine*.

[112] Patole P. S., Gröne H.-J., Segerer S. (2005). Viral double-stranded RNA aggravates lupus nephritis through toll-like receptor 3 on glomerular mesangial cells and antigen-presenting cells. *Journal of the American Society of Nephrology*.

[113] Imaizumi T., Aizawa T., Segawa C. (2014). Toll-like receptor 3 signaling contributes to the expression of a neutrophil chemoattractant, CXCL1 in human mesangial cells. *Clinical and Experimental Nephrology*.

[114] Ka S.-M., Cheng C.-W., Shui H.-A. (2007). Mesangial cells of lupus-prone mice are sensitive to chemokine production. *Arthritis Research & Therapy*.

[115] Hedberg A., Kanapathippillai P., Rekvig O. P., Fenton K. A. (2013). LMW heparin prevents increased kidney expression of proinflammatory mediators in (NZBxNZW)F1 mice. *Clinical and Developmental Immunology*.

[116] Qing X., Pitashny M., Thomas D. B., Barrat F. J., Hogarth M. P., Putterman C. (2008). Pathogenic anti-DNA antibodies modulate gene expression in mesangial cells: involvement of HMGB1 in anti-DNA antibody-induced renal injury. *Immunology Letters*.

[117] Rovin B. H., Lu L., Marsh C. B. (2001). Lymphocytes induce monocyte chemoattractant protein-1 production by renal cells after Fc*γ* receptor cross-linking: role of IL-1*β*. *Journal of Leukocyte Biology*.

[118] Suzuki E., Karam E., Williams S., Watson D. K., Gilkeson G., Zhang X. K. (2012). Fli-1 transcription factor affects glomerulonephritis development by regulating expression of monocyte chemoattractant protein-1 in endothelial cells in the kidney. *Clinical Immunology*.

[119] Lennard Richard M. L., Sato S., Suzuki E., Williams S., Nowling T. K., Zhang X. K. (2014). The Fli-1 transcription factor regulates the expression of CCL5/RANTES. *Journal of Immunology*.

[120] Moreth K., Brodbeck R., Babelova A. (2010). The proteoglycan biglycan regulates expression of the B cell chemoattractant CXCL13 and aggravates murine lupus nephritis. *The Journal of Clinical Investigation*.

[121] Pawar R. D., Patole P. S., Zecher D. (2006). Toll-like receptor-7 modulates immune complex glomerulonephritis. *Journal of the American Society of Nephrology*.

[122] Zhao X., Tang Y., Qu B. (2010). MicroRNA-125a contributes to elevated inflammatory chemokine RANTES levels via targeting KLF13 in systemic lupus erythematosus. *Arthritis & Rheumatism*.

[123] Mohammed M. F., Belal D., Bakry S. (2014). A study of hepcidin and monocyte chemoattractant protein-1 in egyptian females with systemic lupus erythematosus. *Journal of Clinical Laboratory Analysis*.

[124] Watson L., Midgley A., Pilkington C. (2012). Urinary monocyte chemoattractant protein 1 and alpha 1 acid glycoprotein as biomarkers of renal disease activity in juvenile-onset systemic lupus erythematosus. *Lupus*.

[125] Aranda F., Wingeyer S. P., Mũnoz S. A. (2012). The -2518 A/G polymorphism in the monocyte chemoattractant protein 1 gene is associated with the risk of developing systemic lupus erythematosus in argentinean patients: a multicenter study. *European Cytokine Network*.

[126] Marks S. D., Shah V., Pilkington C., Tullus K. (2010). Urinary monocyte chemoattractant protein-1 correlates with disease activity in lupus nephritis. *Pediatric Nephrology*.

[127] Alzawawy A., Zohary M., Ablordiny M., Eldalie M. (2009). Estimation of monocyte-chemoattractantprotein-1 (Mcp-1) level in patients with lupus nephritis. *International Journal of Rheumatic Diseases*.

[128] Chan R. W.-Y., Lai F. M.-M., Li E. K.-M. (2004). Expression of chemokine and fibrosing factor messenger RNA in the urinary sediment of patients with lupus nephritis. *Arthritis and Rheumatism*.

[129] Rovin B. H., Song H., Birmingham D. J., Hebert L. A., Yu C. Y., Nagaraja H. N. (2005). Urine chemokines as biomarkers of human systemic lupus erythematosus activity. *Journal of the American Society of Nephrology*.

[130] Noris M., Bernasconi S., Casiraghi F. (1995). Monocyte chemoattractant protein-1 is excreted in excessive amounts in the urine of patients with lupus nephritis. *Laboratory Investigation*.

[131] Brunner H. I., Bennett M. R., Mina R. (2012). Association of noninvasively measured renal protein biomarkers with histologic features of lupus nephritis. *Arthritis and Rheumatism*.

[132] Singh R. G., Usha, Rathore S. S., Behura S. K., Singh N. K. (2012). Urinary MCP-1 as diagnostic and prognostic marker in patients with lupus nephritis flare. *Lupus*.

[133] Zhang X., Nagaraja H. N., Nadasdy T. (2012). A composite urine biomarker reflects interstitial inflammation in lupus nephritis kidney biopsies. *Kidney International*.

[134] Marie M. A., Abu Khalil R. E., Habib H. M. (2013). Urinary CXCL10: a marker of nephritis in lupus patients. *Reumatismo*.

[135] Avihingsanon Y., Phumesin P., Benjachat T. (2006). Measurement of urinary chemokine and growth factor messenger RNAs: a noninvasive monitoring in lupus nephritis. *Kidney International*.

[136] Watson L., Tullus K., Pilkington C. (2014). Urine biomarkers for monitoring juvenile lupus nephritis: a prospective longitudinal study. *Pediatric Nephrology*.

[137] Abujam B., Cheekatla S. S., Aggarwal A. (2013). Urinary CXCL-10/IP-10 and MCP-1 as markers to assess activity of lupus nephritis. *Lupus*.

[138] Solari R., Pease J. E., Begg M. (2015). Chemokine receptors as therapeutic targets: why aren't there more drugs?. *European Journal of Pharmacology*.

[139] Proudfoot A. E. I., Bonvin P., Power C. A. (2015). Targeting chemokines: pathogens can, why can't we?. *Cytokine*.

[140] Horuk R. (2009). Chemokine receptor antagonists: overcoming developmental hurdles. *Nature Reviews Drug Discovery*.

[141] Schnickel G. T., Bastani S., Hsieh G. R. (2008). Combined CXCR3/CCR5 blockade attenuates acute and chronic rejection. *Journal of Immunology*.

